# Molecular characterization of multidrug-resistant *Mycobacterium tuberculosis* (MDR-TB) isolates identifies local transmission of infection in Kuwait, a country with a low incidence of TB and MDR-TB

**DOI:** 10.1186/s40001-019-0397-2

**Published:** 2019-12-05

**Authors:** Noura M. Al-Mutairi, Suhail Ahmad, Eiman M. Mokaddas

**Affiliations:** Department of Microbiology, Faculty of Medicine, Kuwait University, P. O. Box 24923, 13110 Safat, Kuwait

**Keywords:** *Mycobacterium tuberculosis*, MDR-TB, Resistance mechanisms, Molecular epidemiology, Local transmission

## Abstract

**Background:**

Increasing incidence of multidrug-resistant *Mycobacterium tuberculosis* infections is hampering global tuberculosis control efforts. Kuwait is a low-tuberculosis-incidence country, and ~ 1% of *M. tuberculosis* strains are resistant to rifampicin and isoniazid (MDR-TB). This study detected mutations in seven genes predicting resistance to rifampicin, isoniazid, pyrazinamide, ethambutol and streptomycin in MDR-TB strains. Sequence data were combined with spoligotypes for detecting local transmission of MDR-TB in Kuwait.

**Methods:**

Ninety-three MDR-TB strains isolated from 12 Kuwaiti and 81 expatriate patients and 50 pansusceptible strains were used. Phenotypic drug susceptibility was determined by MGIT 460 TB/960 system. Mutations conferring resistance to rifampicin, isoniazid, pyrazinamide, ethambutol and streptomycin were detected by genotype MTBDR*plus* assay and/or PCR sequencing of three *rpoB* regions*, katG* codon 315 (*katG315*) + *inhA* regulatory region, *pncA*, three *embB* regions and *rpsL* + *rrs*-500–900 regions. Spoligotyping kit was used, spoligotypes were identified by SITVIT2, and phylogenetic tree was constructed by using MIRU-VNTRplus software. Phylogenetic tree was also constructed from concatenated sequences by MEGA7 software. Additional PCR sequencing of *gidB* and *rpsA* was performed for cluster isolates.

**Results:**

Pansusceptible isolates contained wild-type sequences. Mutations in *rpoB* and *katG* and/or *inhA* were detected in 93/93 and 92/93 MDR-TB strains, respectively. Mutations were also detected for pyrazinamide resistance, ethambutol resistance and streptomycin resistance in MDR-TB isolates in *pncA*, *embB* and *rpsL* + *rrs*, respectively. Spoligotyping identified 35 patterns with 18 isolates exhibiting unique patterns while 75 isolates grouped in 17 patterns. Beijing genotype was most common (32/93), and 11 isolates showed nine orphan patterns. Phylogenetic analysis of concatenated sequences showed unique patterns for 51 isolates while 42 isolates grouped in 16 clusters. Interestingly, 22 isolates in eight clusters by both methods were isolated from TB patients typically within a span of 2 years. Five of eight clusters were confirmed by additional *gidB* and *rpsA* sequence data.

**Conclusions:**

Our study provides the first insight into molecular epidemiology of MDR-TB in Kuwait and identified several potential clusters of local transmission of MDR-TB involving 2–6 subjects which had escaped detection by routine surveillance studies. Prospective detection of resistance-conferring mutations can identify possible cases of local transmission of MDR-TB in low MDR-TB settings.

## Background

Tuberculosis (TB) is a major infectious disease of global proportions. According to the annual surveys conducted by World Health Organization (WHO), an estimated 10 million new active TB diseases cases and 1.6 million deaths occurred in 2017, making TB as the leading cause of death from a single infectious agent [1]. Near 87% of all TB cases occurred in 30 high-TB-burden countries with nearly 67% of all cases occurring in only eight (India, China, Indonesia, Philippines, Pakistan, Nigeria, Bangladesh and South Africa) countries. In high-TB-burden countries, active TB disease cases usually occur as a result of recent infection or reinfection while in low-TB-incidence countries, most active disease cases occur as a result of reactivation of latent infection acquired few to several years earlier [2–4]. Most of the TB deaths recorded in recent years have been attributed to drug-resistant (DR) TB [1]. Worldwide, 558,000 people developed TB in 2017 that was resistant to rifampicin, and of these, nearly 457,000 (82%) were inflicted with *Mycobacterium tuberculosis* strains resistant at least to rifampicin and isoniazid, the two most effective first-line drugs (MDR-TB) [1]. The MDR-TB in resource-limited settings is difficult to treat due to lengthy, more expensive and more toxic treatment regimens which lead to higher rates of clinical failure and disease relapse [5, 6]. MDR-TB is also a risk factor for the development of extensively drug-resistant TB (XDR-TB), infection with MDR-TB strains additionally resistant to a fluoroquinolone and an injectable anti-TB drug, which is even more difficult to treat than MDR-TB [1, 5, 6]. Globally, treatment success rates for fully susceptible TB, MDR-TB and XDR-TB have been estimated as nearly 95%, 55% and 28%, respectively [1]. Rapid and accurate laboratory diagnosis of MDR-TB is crucial for effective treatment, which will also limit further transmission of MDR-TB and evolution of XDR-TB [1, 5].

The evolution of drug resistance in clinical *M. tuberculosis* strains is mainly due to chromosomal mutations in target genes [5, 7]. The WHO now recommends that drug susceptibility testing (DST) of *M. tuberculosis* isolates should be carried out for all patients with TB to guide treatment decisions and to improve outcome [1]. Phenotypic DST methods are time-consuming as they require several days to few weeks to report results and are also not widely available [8]. Recent studies have shown that gene sequencing studies reliably predict susceptibility or resistance to first-line anti-TB drugs and are even superior to phenotypic-based DST methods for some first-line drugs [9–13].

Kuwait, an Arabian Gulf country in the Middle East, is a low TB incidence (~ 24 cases/100 000 population) country [14, 15]. The expatriates account for nearly 75% of the total population of Kuwait. Although all expatriates are screened at the time of entry into Kuwait for the absence of active TB disease, > 80% of all TB cases and > 95% of DR-TB and MDR-TB cases occur among expatriate patients, mainly originating from TB endemic countries of South Asia (Bangladesh, India, Pakistan, etc.), Southeast Asia (mainly Philippines and Indonesia) and Africa [16–19]. The diversity of mutations and fingerprinting profiles among multidrug-resistant *M. tuberculosis* isolates have previously shown that most expatriate patients developed active disease in Kuwait due to reactivation of previously acquired infection [17, 18]. Until recently, rifampicin-resistant TB/MDR-TB among Kuwaiti subjects were infrequently detected [16–19] and transmission of DR-TB was rarely reported in Kuwait [20]. A fourfold increase in the detection of MDR-TB among Kuwaiti subjects was noted in recent years (2014–2017); however, the factors responsible for this sudden increase remained unknown and transmission of MDR-TB within Kuwait was not apparent from routine surveillance studies. This study performed detailed molecular characterization of all MDR-TB strains collected during a 12-year period (2006 to 2017) to detect mutations in seven major gene targets (*rpoB*, *katG*, *inhA*, *pncA, embB, rpsL* and *rrs*) conferring resistance to rifampicin, isoniazid, pyrazinamide, ethambutol and streptomycin. Fingerprinting of all isolates was performed by spoligotyping. The sequence data obtained from multiple loci were also used to generate phylogenetic tree to ascertain strain relatedness (multi-locus sequence analysis, MLSA). Data from spoligotyping and MLSA were combined to define cluster(s) of isolates possibly reflecting local transmission of infection within Kuwait. Sequence data for two additional loci (*gidB* and *rpsA*) known to carry phylogenetic polymorphisms [21, 22] were also obtained for cluster isolates obtained within a short time frame (nearly 2 years) to confirm their close genetic relatedness.

## Methods

### Patients, specimens and *M. tuberculosis* isolates

A total of 93 multidrug-resistant *M. tuberculosis* (MDR-TB) strains obtained from 93 suspected TB patients during 2006 to 2017 and 50 drug-susceptible *M. tuberculosis* isolates collected from 50 TB patients were analyzed in this study. The MDR-TB strains were cultured from 74 pulmonary and 19 extra-pulmonary specimens at Kuwait National TB Control Laboratory (KNTCL). All clinical specimens were collected from suspected TB patients before initiation of treatment with anti-TB drugs and after obtaining verbal consent as part of routine patient care, diagnostic workup and resistance surveillance. All specimens were cultured on solid (Lowenstein-Jensen) and liquid (mycobacteria growth indicator tube, MGIT 960 system) media according to manufacturer’s instructions (Becton Dickinson, Sparks, MD, USA) and as described previously [14, 23]. Repeat isolates typically cultured within 1 week of isolation of the first isolate were also available from 35 patients yielding MDR-TB strains. The MGIT 960 system cultures were analyzed at Reference Mycobacteriology Laboratory, Department of Microbiology, Faculty of Medicine, Kuwait University, and the results are reported in this paper anonymously, without revealing patient identity.

Non-sterile samples were processed by using *N*-acetyl-l-cysteine and sodium hydroxide (NALC/NaOH), while sterile clinical specimens were processed directly for the cultivation of mycobacteria, as described previously [14, 23]. All samples yielded a positive growth reading in MGIT 960 system cultures, and all MGIT cultures were positive for the presence of acid-fast bacilli by Ziehl-Neelsen smear microscopy and for *M. tuberculosis* complex DNA by AccuProbe DNA probe assay and by an in-house multiplex PCR assay, performed as described previously [23, 24].

### Phenotypic drug susceptibility testing

All *M. tuberculosis* isolates were subjected to phenotypic DST against rifampicin, isoniazid, ethambutol and streptomycin by using Bactec 460 TB system (for isolates collected during 2006–2010) or MGIT 960 system (for isolates collected during 2011–2017) using SIRE drug kit, according to the manufacturer’s recommendations and as described in detail previously [14, 23]. The DST against pyrazinamide (PZA) was also performed for the isolates by using MGIT 960 system and the MGIT 960 PZA kits according to the manufacturer’s instructions (Becton Dickinson).

### Molecular characterization for detection of mutations predicting resistance

Genomic DNA was extracted from each MGIT culture by the rapid Chelex-100 method as described in detail previously [25]. The MDR-TB status of all isolates was tested by the GenoType MTBDR*plus* line probe assay, performed as described previously [26]. The results were confirmed and extended by PCR amplification followed by DNA sequencing (PCR sequencing) of three (hot spot, N-terminal and cluster II) regions of *rpoB* gene, *katG* codon 315 (*katG315*) and *inhA* regulatory region (*inhA*-RR) by using gene-specific PCR (Additional file 1: Table S1) and sequencing (Additional file 2: Table S2) primers and as described previously [26]. For isolates with wild-type sequence for *katG315* and *inhA*-RR, the extended N-terminal regions of *katG* and *inhA* were sequenced by using gene-specific PCR (Additional file 1: Table S1) and sequencing (Additional file 2: Table S2) primers. The molecular basis of resistance to ethambutol, streptomycin and pyrazinamide was also determined, as described previously, by PCR sequencing of *embB306* + *embB406* + *embB497* regions [27, 28], *rpsL* + *rrs* (500 and 900 regions) [28] and *pncA* gene [12, 29], respectively, by using gene-specific PCR (Additional file 1: Table S1) and sequencing (Additional file 2: Table S2) primers. The sequence data for *rpoB*, *katG*, *inhA*, *pncA*, *embB*, *rpsL* and *rrs* loci obtained for each isolate were concatenated and the combined sequence data were used to construct phylogenetic tree by using the unweighted pair group method with arithmetic averages (UPGMA) settings by using MEGA7 software. A cluster was defined when two or more patient isolates shared the same sequence profile for all seven loci.

### Spoligotyping

All MDR-TB isolates were also subjected to spoligotyping, performed as described previously [30]. The results in binary format were used for assignment of phylogenetic lineages according to SITVIT database (http://www.pasteur-guadeloupe.fr.:8081/SITVITDemo/index.jsp). The spoligotype-based dendrogram was generated by UPGMA calculation using MIRU-VNTRplus web page (http://www.miru-vntrplus.org). A cluster was defined when two or more patient isolates shared same spoligotype pattern. Spoligotype patterns not described in SITVIT2 database were designated as ‘orphan’ [31].

### Further molecular analysis of cluster isolates

PCR sequencing of *gidB* and *rpsA*, *M. tuberculosis* targets that may confer resistance to streptomycin and pyrazinamide, respectively, and known to carry lineage-specific polymorphisms [21, 22, 32] was also carried out for isolates clustered by both (multi-gene sequence data and spoligotyping data) fingerprinting methods and were obtained from TB patients within a 2-year period by using gene-specific PCR (Additional file 2: Table S1) and sequencing (Additional file 2: Table S2) primers and the PCR amplification and sequencing protocols described previously [23, 26]. Additional file 1: Table S1 showing primer sequences for PCR amplification while Additional file 2: Table S2 showing primer sequences used for DNA sequencing of various gene loci.

### Statistical analysis

Categorical variables were expressed as absolute number. Statistical analysis was performed using Pearson’s Chi square test and probability levels < 0.05 were considered as statistically significant. Statistical analyses were performed by using WinPepi software ver. 11.65 (PEPI for Windows, Microsoft Inc., Redmond, WA, USA).

## Results

### Characteristics of *M. tuberculosis* isolates

A total of 93 MDR-TB strains obtained from 93 TB patients during 2006 to 2017 (representing all available MDR-TB strains collected during this period) and 50 fully susceptible *M. tuberculosis* isolates collected from 50 TB patients were used. The MDR-TB strains were cultured from 74 pulmonary (sputum, *n* = 66 and bronchoalveolar lavage, *n* = 8) and 19 extra-pulmonary (fine needle aspirate, *n* = 8; pus, *n* = 5; tissue biopsy, *n* = 3; lymph node, *n* = 2 and cerebrospinal fluid, *n* = 1) specimens obtained from 12 Kuwaiti and 81 expatriate (Indian, *n* = 35; Ethiopian, *n* = 15; Filipino, *n* = 13; Iraqi, *n* = 4; Nepalese, *n* = 4; Egyptian, *n* = 3; Bangladeshi, *n* = 2; Indonesian, *n* = 2; Syrian, *n* = 1; Saudi Arabian, *n* = 1 and Georgian, *n* = 1) patients (males *n* = 49; females, *n* = 44). All isolates were cultured from newly diagnosed TB patients before initiation of treatment with anti-TB drugs. Prior treatment history was not available for expatriate TB patients.

The 50 drug-susceptible *M. tuberculosis* isolates were susceptible to all four (rifampicin, isoniazid, ethambutol and streptomycin) drugs tested (pansusceptible strains). All 93 drug-resistant isolates were uniformly resistant to rifampicin and isoniazid (MDR-TB strains). Forty-one isolates were additionally resistant to ethambutol while 59 isolates were additionally resistant to streptomycin (Table 1). Although all 93 MDR-TB isolates were tested for susceptibility to pyrazinamide, only 47 isolates yielded interpretable results; 11 isolates were susceptible, and 36 were resistant to this drug including 15 isolates that were resistant to all five drugs. The remaining 46 MDR-TB strains failed to grow at the reduced pH in the absence of the drug. No resistance-conferring mutation was detected in any of the seven target genes/gene fragments in any of the 50 pansusceptible strains analyzed in this study. The proportion of MDR-TB isolates exhibiting resistance-conferring mutations in target genes varied for different anti-TB drugs, being highest for rifampicin and lowest for streptomycin (Table 1). Compared to rifampicin, the differences were statistically significant for ethambutol (*P* = 0.008) and streptomycin (*P* = 0.000). Repeat MDR-TB isolates, cultured from 35 patients within 1 week of isolation of the first isolate, yielded the same resistance and mutation patterns as the first isolate from each patient.

**Table 1:** Phenotypic resistance by MGIT 960 system to anti-TB drugs among 93 multidrug-resistant *M. tuberculosis* isolates and number of susceptible and resistant isolates with mutations in target genes for each drug

Anti-tuberculosis	No. of isolates	No. of susceptible	No. of susceptible	No. of resistant	No. (%) of resistant
Drug	Tested	Isolates	Isolates with mutation^a^	Isolates	Isolates with mutation^a^
Rifampicin	93	0	0	93	93 (100)
Isoniazid	93	0	0	93	92 (98.9)
Pyrazinamide	47	11	0	36	30 (83.3)
Ethambutol	93	52	38^b^	41	38 (92.7)
Streptomycin	93	34	0	59	49 (83.1)

^a^Resistance-conferring mutations were detected in *rpoB* for rifampicin, *katG* + *inhA* for isoniazid, *pncA* for pyrazinamide, *embB* for ethambutol, and *rpsL* + *rrs* for streptomycin

^b^*Mycobacterium tuberculosis* isolates with *embB* mutations usually confer low level of resistance to ethambutol which are often missed by the MGIT 960 system [23, 28]

### Molecular detection of mutations predicting resistance in MDR-TB strains

The combination of GenoType MTBDR*plus* assay and PCR sequencing of *rpoB*, *katG* and *inhA* identified *rpoB* mutations (S456L, *n* = 66; H451Y, *n* = 6, D441V, *n* = 4; H451D, *n* = 3; S456W, *n* = 3; Q438K, *n* = 2; V176F, *n* = 2; Q438E, *n* = 1; Q438P, *n* = 1; H451R, *n* = 1; M440I + D441Y, *n* = 2 and D441V + H451Q, *n* = 1) in all 93 isolates and *katG* and *inhA* mutations in 92 (*katG* S315T, *n* = 74; *inhA*-RR −15 C/T, *n* = 11; *katG* S315T + *inhA*-RR −15 C/T, *n* = 3; *katG* S315N, *n* = 1; *inhA* S94A, *n* = 1; *katG* S315T + *inhA*-RR −8 T/A, *n* = 1 and *katG* S315T + *inhA*-RR −17 G/T, *n* = 1) of 93 isolates. The occurrence of the most common *rpoB* (S456L) mutation was nearly same in MDR-TB strains resistant to only two drugs (18 of 27) versus three drugs (24 of 32) (*P* = 0.481) or versus all four SIRE drugs (24 of 34) (*P* = 0.743). The occurrence of S315T mutation in *katG* was significantly lower in MDR-TB strains resistant to two drugs versus isolates resistant to three drugs (19 of 27 versus 29 of 32, *P* = 0.047) as well as versus isolates resistant to all four drugs (19 of 27 versus 31 of 34, *P* = 0.036). On the contrary, the occurrence of *inhA*-RR −15 C/T mutation alone was significantly higher in MDR-TB strains resistant to two drugs versus isolates resistant to three drugs (7 of 27 versus 2 of 32, *P* = 0.036) as well as versus isolates resistant to all four drugs (7 of 27 versus 2 of 34, *P* = 0.028).

PCR sequencing of *pncA* identified mutations in 30 of 36 MDR-TB strains phenotypically resistant to pyrazinamide and 23 of 46 isolates for which phenotypic DST data for pyrazinamide was not available while all 11 isolates phenotypically susceptible to pyrazinamide contained wild-type sequence for *pncA*. The *pncA* mutations included −11 A/G (*n* = 13), non-synonymous mutations (*n* = 28) spread over 19 codon positions; insertion frame shift mutations (*n* = 6); insertion of GGT at nucleotide 390 (*n* = 1); deletion frame shift mutation (*n* = 1); premature termination at codon 111 (*n* = 1); −7 T/C (*n* = 1); −15 A/C + V130A (*n* = 1) and conversion of initiation codon ATG to CTG (*n* = 1). Synonymous mutations (TCC65TCT, *n* = 15 and TCC65TCG, *n* = 2) considered as phylogenetic markers were also identified in 17 isolates.

As reported earlier [23, 28], *embB* mutations were detected in both ethambutol-resistant and ethambutol-susceptible MDR-TB strains (Table 1). Fifty isolates contained mutations at *embB306* (M306V, *n* = 28; M306I, *n* = 19 and M306L, *n* = 3), 15 isolates contained a mutated *embB406* (G406D, *n* = 8; G406A, *n* = 4; G406C, *n* = 2 and G406S, *n* = 1), 10 isolates contained a mutated *embB497* (Q497R, *n* = 6; Q497K, *n* = 3 and Q497H, *n* = 1) and one isolate contained a mutation (Y319S) at *embB319*. Other mutations (L355L, CTG355CTA + E378A, GAG378GCG, *n* = 3 and E378A, *n* = 2) considered as phylogenetic markers not related with resistance to ethambutol [28] were also identified in five isolates. Forty-nine of 59 MDR-TB strains additionally resistant to streptomycin contained a mutation in the target genes analyzed (Table 1), many of which have been described previously [23, 28]. These included 44 isolates with a mutation in *rpsL* (K43R, *n* = 33; K43T, *n* = 1; K88R, *n* = 5; K88T, *n* = 4; K88M, *n* = 1), four isolates with a mutation in *rrs* 500 or 900 region (A514C, *n* = 1; C517T, *n* = 1; G878A, *n* = 1 and A906G, *n* = 1) and one isolate with *rpsL* K88R + *rrs* C602A double mutation.

### Spoligotyping analyses

**Fig. 1:**
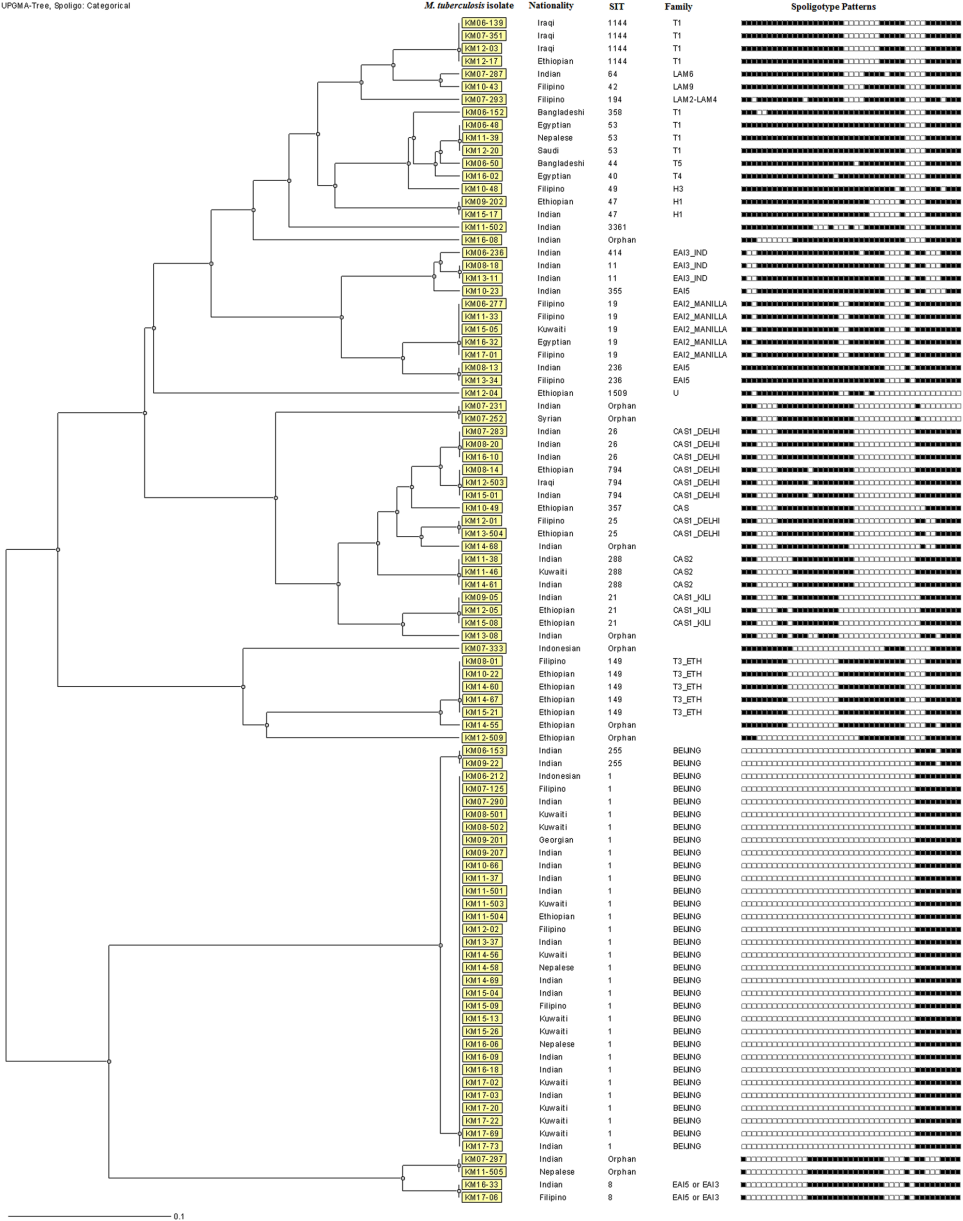
The dendrogram generated from the spoligotyping data using MIRU-VNTRplus database and unweighted pair group method with arithmetic mean (UPGMA) settings for 93 multidrug-resistant *M. tuberculosis* isolates from Kuwait is shown. The nationality of the TB patient yielding the isolate, shared international type (SIT), *M. tuberculosis* family (Family) and the actual spoligotyping patterns for each isolate are shown in vertical columns

Initial fingerprinting performed by spoligotyping showed that 82 of 93 MDR-TB strains belonged to specific shared international type (SIT), while 11 isolates showed nine unique (orphan) patterns that were not found in SITVIT2 database. The dendrogram generated from the spoligotyping data showed that 18 isolates exhibited unique patterns while 75 isolates grouped in 17 clusters with each cluster containing 2–30 isolates (Fig. 1). The SIT1 pattern was most common (*n* = 30), and Beijing was the most common family shared among 32 isolates. The occurrence of Beijing genotype was significantly higher in MDR-TB strains resistant to all four drugs versus isolates resistant to two drugs (18 of 34 versus 4 of 27, *P* = 0.002) but not versus isolates resistant to three drugs (18 of 34 versus 10 of 32, *P* = 0.075). Interestingly, 10 of 12 Kuwaiti patients were infected with Beijing genotype.

### Fingerprinting based on multi-locus sequence analysis

**Fig. 2:**
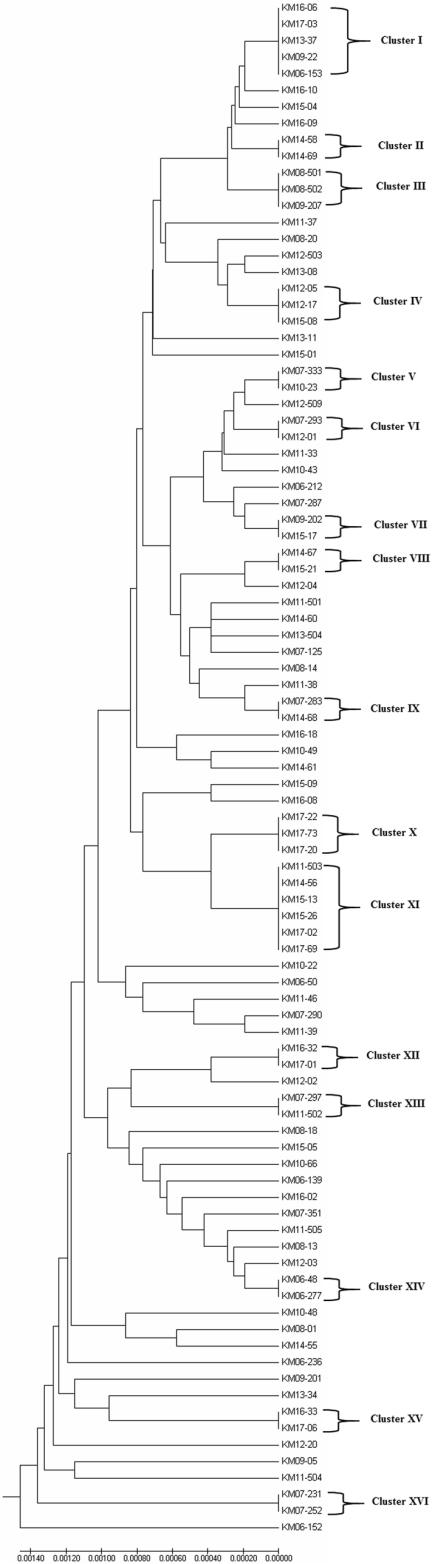
The dendrogram obtained from concatenated (complete or partial) sequence data for seven (*rpoB, katG, inhA, pncA, embB, rpsL* and *rrs*) loci generated by using MEGA7 phylogenetic software with unweighted pair group method with arithmetic mean (UPGMA) settings for 93 multidrug-resistant *M. tuberculosis* isolates from Kuwait is shown. Isolates with identical sequence patterns were classified as cluster isolates and all 16 clusters (Cluster I to Cluster XVI) are marked

The DNA sequence data generated from the partial or complete sequencing of seven (*rpoB*, *katG*, *inhA*, *pncA*, *embB*, *rpsL* and *rrs*) loci was also used to determine phylogenetic relationship among the 93 MDR-TB strains. The dendrogram based on concatenated sequence data showed 51 isolates with unique sequences, while 42 isolates grouped in 16 clusters (labeled as Cluster I to Cluster XVI) with each cluster containing 2–6 isolates (Fig. 2). The MLSA data were combined with data obtained from spoligotyping, time interval between isolation of cluster isolates with/without additional sequence data for two other (*gidB* and *rpsA*) loci and the results are presented in Table 2. Based on spoligotyping data, both isolates in five clusters (Cluster V, VI, IX, XIII and XIV) were genetically unrelated strains and/or were isolated more than 2 years apart. Similarly, isolates in three other clusters were either genetically heterogeneous (Cluster 1 and IV) or were isolated 6 years apart (Cluster VII) and so were not considered epidemiologically related strains. On the contrary, isolates in eight clusters (Cluster II, III, VIII, X, XI, XII, XV and XVI) exhibited the same spoligotype and were obtained within a short time span (nearly 2 years). Further analysis of *gidB* and *rpsA* sequence data showed that all isolates in five clusters (Cluster III, VIII, XI, XV and XVI) were genotypically identical strains, highly suggestive of cross-transmission of infection among these patients or infection from a common source. Also, two of three isolates in Cluster X were identical while the two isolates in Cluster XII were very closely related. On the contrary, the two isolates in Cluster II were genotypically unrelated strains (Table 2).

**Table 2:** Detailed clinical, demographic and molecular characteristics of 42 *M. tuberculosis* isolates in 16 (Cluster I to Cluster XVI) clusters

Cluster no.	Clinical specimen	Isolate no.	Year of isolation	Patient’s nationality	Spoligotyping data		Genetic alteration detected in								
					SIT	Mtb family	*rpoB*	*katG*	*inhA*	*pncA*	*embB*	*rpsL*	*rrs*	*gidB*	*rpsA*
I	Sputum	KM06-153	2006	Indian	255	Beijing	TCG456TTG	ACG315ACC	WT	WT	ATG306GTG	AAG43AGG	WT	N. D.	N. D.
	CSF	KM09-22	2009	Indian	255	Beijing	TCG456TTG	ACG315ACC	WT	WT	ATG306GTG	AAG43AGG	WT	N. D.	N. D.
	Sputum	KM13-37	2013	Indian	1	Beijing	TCG456TTG	ACG315ACC	WT	WT	ATG306GTG	AAG43AGG	WT	N. D.	N. D.
	FNA	KM16-06	2016	Nepalese	1	Beijing	TCG456TTG	ACG315ACC	WT	WT	ATG306GTG	AAG43AGG	WT	N. D.	N. D.
	FNA	KM17-03	2017	Indian	1	Beijing	TCG456TTG	ACG315ACC	WT	WT	ATG306GTG	AAG43AGG	WT	N. D.	N. D.
II	Sputum	KM14-58	2014	Nepalese	1	Beijing	TCG456TTG	ACG315ACC	WT	GTG139GCG	ATG306GTG	AAG43AGG	WT	GAA92GAC + *GCA205GCG*	*CGA212CGC*
	Sputum	KM14-69	2014	Indian	1	Beijing	TCG456TTG	ACG315ACC	WT	GTG139GCG	ATG306GTG	AAG43AGG	WT	*GCA205GCG*	WT
III	Sputum	KM08-501	2008	Kuwaiti	1	Beijing	TCG456TTG	ACG315ACC	WT	GGT139GTT	ATG306GTG	AAG43AGG	WT	GAA92GAC + *GCA205GCG*	*CGA212CGC*
	Sputum	KM08-502	2008	Kuwaiti	1	Beijing	TCG456TTG	ACG315ACC	WT	GGT139GTT	ATG306GTG	AAG43AGG	WT	GAA92GAC + *GCA205GCG*	*CGA212CGC*
	Sputum	KM09-207	2009	Indian	1	Beijing	TCG456TTG	ACG315ACC	WT	GGT139GTT	ATG306GTG	AAG43AGG	WT	GAA92GAC + *GCA205GCG*	*CGA212CGC*
IV	Sputum	KM12-05	2012	Ethiopian	21	CAS1-Kili	TCG456TTG	ACG315ACC	WT	Ins193A (FS) + *TCC65TCT*	ATG306GTG	AAG88AGG	WT	N. D.	N. D.
	Sputum	KM12-17	2012	Ethiopian	1144	T1	TCG456TTG	ACG315ACC	WT	Ins193A (FS) + *TCC65TCT*	ATG306GTG	AAG88AGG	WT	N. D.	N. D.
	Sputum	KM15-08	2015	Ethiopian	21	CAS1-Kili	TCG456TTG	ACG315ACC	WT	Ins193A (FS) + *TCC65TCT*	ATG306GTG	AAG88AGG	WT	N. D.	N. D.
V	Sputum	KM07-333	2007	Indonesian	Orphan	N. A.	TCG456TTG	ACG315ACC	WT	WT	WT	WT	WT	N. D.	N. D.
	Sputum	KM10-23	2010	Indian	355	EAI3-IND	TCG456TTG	ACG315ACC	WT	WT	WT	WT	WT	N. D.	N. D.
VI	Sputum	KM07-293	2007	Filipino	194	LAM2	TCG456TTG	ACG315ACC	WT	WT	CAG497CGG	WT	WT	N. D.	N. D.
	Sputum	KM12-01	2012	Filipino	25	CAS1-Delhi	TCG456TTG	ACG315ACC	WT	WT	CAG497CGG	WT	WT	N. D.	N. D.
VII	Sputum	KM09-202	2009	Ethiopian	47	H1	GTC176TTC	ACG315ACC	WT	WT	WT	WT	WT	N. D.	N. D.
	Sputum	KM15-17	2015	Indian	47	H1	GTC176TTC	ACG315ACC	WT	WT	WT	WT	WT	N. D.	N. D.
VIII	Sputum	KM14-67	2014	Ethiopian	149	T3-ETH	TCG456TTG	ACG315ACC	WT	−11 A/G	ATG306ATC	WT	WT	GGT69GAT	WT
	Sputum	KM15-21	2015	Ethiopian	149	T3-ETH	TCG456TTG	ACG315ACC	WT	−11 A/G	ATG306ATC	WT	WT	GGT69GAT	WT
IX	Sputum	KM07-283	2007	Indian	26	CAS1-Delhi	TCG456TTG	ACG315ACC	WT	*TCC65TCT*	ATG306ATA	WT	WT	N. D.	N. D.
	Sputum	KM14-68	2014	Indian	Orphan	N. A.	TCG456TTG	ACG315ACC	WT	*TCC65TCT*	ATG306ATA	WT	WT	N. D.	N. D.
	Sputum	KM17-20	2017	Kuwaiti	1	Beijing	TCG456TTG	ACG315ACC	WT	−11 A/G	CAG497CGG	AAG43AGG	WT	GAA92GAC + *GCA205GCG*	WT
X	Sputum	KM17-22	2017	Kuwaiti	1	Beijing	TCG456TTG	ACG315ACC	WT	−11 A/G	CAG497CGG	AAG43AGG	WT	GAA92GAC + *GCA205GCG*	*CGA212CGC*
	Sputum	KM17-73	2017	Indian	1	Beijing	TCG456TTG	ACG315ACC	WT	−11 A/G	CAG497CGG	AAG43AGG	WT	GAA92GAC + *GCA205GCG*	*CGA212CGC*
	Pus	KM11-503	2011	Kuwaiti	1	Beijing	TCG456TTG	ACG315ACC	WT	−11 A/G	GGC406GAC	AAG43AGG	WT	GAA92GAC + GCA205GCG	*CGA212CGC*
	Sputum	KM14-56	2014	Kuwaiti	1	Beijing	TCG456TTG	ACG315ACC	WT	−11 A/G	GGC406GAC	AAG43AGG	WT	GAA92GAC + GCA205GCG	*CGA212CGC*
XI	Sputum	KM15-13	2015	Kuwaiti	1	Beijing	TCG456TTG	ACG315ACC	WT	−11 A/G	GGC406GAC	AAG43AGG	WT	GAA92GAC + GCA205GCG	*CGA212CGC*
	Sputum	KM15-26	2015	Kuwaiti	1	Beijing	TCG456TTG	ACG315ACC	WT	−11 A/G	GGC406GAC	AAG43AGG	WT	GAA92GAC + *GCA205GCG*	*CGA212CGC*
	Sputum	KM17-02	2015	Kuwaiti	1	Beijing	TCG456TTG	ACG315ACC	WT	−11 A/G	GGC406GAC	AAG43AGG	WT	GAA92GAC + *GCA205GCG*	*CGA212CGC*
	Sputum	KM17-69	2017	Kuwaiti	1	Beijing	TCG456TTG	ACG315ACC	WT	−11 A/G	GGC406GAC	AAG43AGG	WT	GAA92GAC + *GCA205GCG*	*CGA212CGC*
XII	Sputum	KM16-32	2016	Egyptian	19	EAI2-Manila	CAC451TAC	ACG315ACC	−15 C/T	GAA37AAA	*CTG355CTA* + GAG378GCG	WT	WT	*GTG110GTT* + *GCA205GCG*	WT
	Sputum	KM17-01	2017	Filipino	19	EAI2-Manila	CAC451TAC	ACG315ACC	−15 C/T	GAA37AAA	*CTG355CTA* + GAG378GCG	WT	WT	CTC95TTC + *GTG110GTT* + *GCA205GCG*	WT
XIII	Pus	KM07-297	2007	Indian	Orphan	N. A.	CAC451GAC	WT	−15 C/T	*TCC65TCG* + Ins 453T (FS)	ATG306CTG	WT	WT	N. D.	N. D.
	FNA	KM11-502	2015	Indian	3361	T1	CAC451GAC	WT	−15 C/T	*TCC65TCG* + Ins 453T (FS)	ATG306CTG	WT	WT	N. D.	N. D.
XIV	Sputum	KM06-48	2006	Egyptian	53	T1	TCG456TTG	WT	−15 C/T	WT	WT	WT	WT	N. D.	N. D.
	Tissue	KM06-277	2006	Filipino	19	EAI2-Manila	TCG456TTG	WT	−15 C/T	WT	WT	WT	WT	N. D.	N. D.
XV	Sputum	KM16-33	2016	Indian	8	EAI3/EAI5	CAC451TAC	ACG315ACC	WT	CTG35CCG	ATG306GTG + GAG378GCG	AAG43AGG	WT	*GTG110GTT* + *GCA205GCG*	WT
	Sputum	KM17-06	2017	Filipino	8	EAI3/EAI5	CAC451TAC	ACG315ACC	WT	CTG35CCG	ATG306GTG + GAG378GCG	AAG43AGG	WT	*GTG110GTT* + *GCA205GCG*	WT
XVI	Sputum	KM07-231	2007	Indian	Orphan^a^	CAS1-Delhi	ATG440ATA + GAC441TAC	ACG315ACC	WT	*TCC65TCT*	GGC406TGC	WT	WT	*GCA205GCG* + Del 350G (FS)	WT
	Sputum	KM07-252	2007	Syrian	Orphan^a^	CAS1-Delhi	ATG440ATA + GAC441TAC	ACG315ACC	WT	*TCC65TCT*	GGC406TGC	WT	WT	*GCA205GCG* + Del 350G (FS)	WT

Clusters containing MDR-TB strains with identical patterns and isolated within a period of nearly 2 years are underlined. Synonymous mutations are italicized

N. A., not applicable; N. D., not done; CSF, cerebrospinal fluid; FNA, fine needle aspirate; SIT, shared international type; Mtb family, *M. tuberculosis* family; WT, wild-type sequence; Ins, insertion mutation; (FS), frame shift mutation

^a^Both isolates displayed identical spoligotyping pattern

## Discussion

The TB control in low incidence settings involves molecular fingerprinting of *M. tuberculosis* isolates to detect unsuspected outbreaks which also indicates possible epidemiological links between TB patients and differentiates reactivated TB from newly acquired infection [1, 33]. Molecular fingerprinting techniques for *M. tuberculosis* have evolved from IS*6110*-based restriction fragment length polymorphism to mycobacterial interspersed repetitive unit-variable-number tandem repeat (MIRU-VNTR) typing with/without spoligotyping and more recently, to whole genome sequence (WGS) comparisons [34–38]. Since whole genome comparisons are not yet standardized, core genome multi-locus sequence typing (cgMLST) has been developed to facilitate high-resolution fingerprinting of clinical *M. tuberculosis* isolates to trace recent transmission of infections [38, 39]. Genomic sequence-based scanning for drug resistance-associated mutations has also been shown to be nearly comparable to MIRU-VNTR typing [40].

More than 80% of all TB cases and > 95% of DR-TB and MDR-TB cases in Kuwait occur among expatriates [14–19]. Unexpectedly, several cases of MDR-TB were diagnosed among Kuwaiti subjects during recent (2014–2017) years, and it was not known whether any of these also resulted from cross-transmission of infection or infection from a common source. In this retrospective study, we performed molecular characterization of all available MDR-TB strains collected over a 12-year period to catalog mutations conferring resistance to first-line drugs to determine the feasibility of using commercial molecular methods for rapid diagnosis of MDR-TB in Kuwait. We also employed sequence-based scanning of seven loci to determine relatedness among MDR-TB strains. Further fingerprinting by spoligotyping combined with sequence data for additional loci known to display phylogenetic polymorphisms and other relevant epidemiological information were used to track transmission of MDR-TB among TB patients in Kuwait.

Our data showed that 91 of 93 (97.8%) MDR-TB strains contained a mutation in *rpoB* hot spot region and S456L mutation was most common, found in 66 of 93 (71%) isolates which is consistent with published reports from different geographical settings [7, 41–43]. Only three MDR-TB strains contained double *rpoB* mutations which is also in line with limited previous exposure of TB patients in Kuwait to anti-TB drugs [41, 43]. Similarly, mutations at *katG315* and *inhA*-RR were detected in 80 of 93 (86.0%) and 16 of 93 (17.2%) MDR-TB strains, respectively, which are consistent with the reported worldwide occurrence of these mutations [7, 41–43]. Altogether, 91 of 93 (98%) MDR-TB strains contained a mutation at *katG315* and/or *inhA*-RR. Of the remaining two isolates, one isolate contained S94A mutation in *inhA* which is known to confer resistance to isoniazid [44]. Taken together, our data show that rapid molecular diagnostic tests (such as GenoType MTBDR*plus* assay and targeted PCR sequencing) that interrogate *rpoB* hot spot region and *katG315* + *inhA*-RR will have a sensitivity of 98% for rifampicin resistance detection, 98% for isoniazid resistance detection and 96% for MDR-TB in Kuwait with 100% specificity. The higher occurrence of S456L mutation in *rpoB* and S315T mutation in *katG* in our MDR-TB strains is also consistent with the low fitness cost associated with these specific mutations [45].

Ethambutol, a first-line drug, may be included in treatment regimens for MDR-TB provided the isolate is susceptible to this drug. Streptomycin is no longer considered a first-line drug and is usually not used in treatment regimens due to high rates of resistance in *M. tuberculosis* isolates globally [5, 6, 46]. Resistance-conferring mutations in *rpsL* and/or *rrs* gene was detected in majority (49 of 59, 83%) of streptomycin-resistant but not in any streptomycin-susceptible MDR-TB strain, while mutations in *embB* gene were detected in both ethambutol-resistant and ethambutol-susceptible MDR-TB strains, as described in our previous studies [23, 28].

Pyrazinamide is used for the treatment of both drug-susceptible TB and MDR-TB [5, 6, 46]. The in vitro susceptibility testing of pyrazinamide is technically challenging and often unreliable due to well-known problems (size of the test inoculum, no growth of some strains in drug-free tube under acidic conditions etc.) recognized in previous studies [47–49]. Phenotypic DST results for pyrazinamide were available for only 47 of 93 MDR-TB strains, while the remaining 46 isolates failed to grow at lower pH. No *pncA* mutation was detected in 50 pansusceptible strains. Analysis of 93 MDR-TB strains showed that 30 of 36 MDR-TB strains phenotypically resistant to pyrazinamide and 23 of 46 isolates for which DST data for pyrazinamide was not available contained a mutation in *pncA* while all 11 MDR-TB strains phenotypically susceptible to pyrazinamide contained wild-type sequence for *pncA*. The −11 A/G mutation in the regulatory region was most common which is known to reduce the expression of pyrazinamidase [29, 50]. Seven isolates contained insertion/deletion frame shift mutations which will also lead to an inactive pyrazinamidase. Non-synonymous, high/very high confidence resistance mutations with/without additional mutation in regulatory region were observed in 29 isolates which have also been detected in other studies [7, 29, 49]. Our data showing a resistance-conferring mutation in 30 of 36 (83%) of pyrazinamide-resistant and in none of 11 pyrazinamide-susceptible MDR-TB strains support previous observations that *pncA* mutations may be used as a surrogate marker of pyrazinamide resistance in *M. tuberculosis* [7, 29, 41, 50, 51].

Spoligotyping data showed that Beijing genotype was most common, shared by 32 of 93 (34%) MDR-TB strains in Kuwait which is expected since this family is known to be strongly associated with multidrug resistance [52]. The occurrence of this genotype was significantly higher in isolates resistant to all four SIRE drugs. The Beijing genotype is also common among drug-resistant/MDR-TB strains from neighboring countries [53, 54]. The occurrence of nine unique (orphan) spoligotyping patterns not reported previously reflects genetic diversity among MDR-TB strains in Kuwait which is not surprising since 81 of 93 (87%) MDR-TB strains were obtained from expatriate patients originating from 11 different countries who were TB-free at the time of their entry into Kuwait, thereby implying that most of these cases likely represent reactivation TB.

The phylogenetic tree generated from concatenated sequence data from partial or complete sequencing of seven (*rpoB*, *katG*, *inhA*, *pncA*, *embB*, *rpsL* and *rrs*) loci identified 42 isolates in 16 clusters (labeled as Cluster I to Cluster XVI) which could likely represent cross-transmission of infection or infection from a common source. This approach has previously been shown to be nearly comparable to the 24-loci-based MIRU-VNTR typing [40, 55, 56]. The first case in each cluster was considered as the ‘index case’ and an arbitrary window period of nearly 2 years was used to define epidemiologically related strains since reactivation of latent infection usually occurs during the first 2 years (even though this period of latency could also be much longer) after infection with *M. tuberculosis* [2]. The two isolates in five clusters (Cluster V, VI, IX, XIII and XIV) were genetically unrelated strains by spoligotyping. Similarly, at least some of the isolates in Cluster I and Cluster IV were genetically unrelated strains by spoligotyping while the two isolates in Cluster VII were separated by a much longer time (6 years) than is normally expected. However, isolates in eight clusters (Cluster II, III, VIII, X, XI, XII, XV and XVI) belonged to the same spoligotype and were obtained within a short time span (nearly 2 years) from each other.

Further analysis of *gidB* and *rpsA* carried out for selected cluster isolates showed that all isolates in five clusters (Cluster III, VIII, XI, XV and XVI) were genotypically identical strains, highly suggestive of cross-transmission of infection among these patients or infection from a common source. Also, two of three isolates in Cluster X were identical. The two isolates in Cluster XII were also very closely related, with the second isolate (KM17-01) displaying an additional mutation (L95F) in *gidB* which is considered as a hot spot for mutations in the *M. tuberculosis* genome [21, 57]. In a recent study, Appelgren et al. [58] investigated transmission of pre-XDR *M. tuberculosis* infection in a healthcare worker in France by gene-scanning of multiple loci involved in conferring resistance to anti-TB drugs and MIRU-VNTR typing. The authors concluded that the healthcare worker had contacted the infection from a patient that displayed nearly identical mutation and MIRU-VNTR patterns. More elaborate sequence comparisons derived from WGS or cgMLST have also identified transmission of infection in low-TB-incidence settings [59–61].

Due to the retrospective nature of our study, investigations to confirm the transmission of infection and identification of epidemiological links and contact tracing were delayed and/or were unsuccessful as most of the TB patients, being expatriate subjects, had left the country, after initial treatment objective (sputum smear-negative status) was achieved. The epidemiological investigations for Kuwaiti patients in Cluster XI were also incomplete as the first case did not appear to be the index case as this patient had extra-pulmonary TB and no obvious connection was apparent between the six subjects during contact tracing. It is probable that the index case was an expatriate patient who is not included in this study and had left the country before the cases described here were characterized. Even WGS comparisons sometimes fail to identify transmission of TB in a setting with a high proportion of migrant (expatriate) cases [62].

Our study has a few limitations. (i) Due to retrospective nature of the study, all MDR-TB isolates collected during the study period were not included and epidemiological investigations and contact tracing were delayed. Since expatriate TB patients are sent back to their respective country after initial treatment objective (sputum smear-negative status) was achieved, some patients could not be contacted as they had left the country. (ii) Molecular fingerprinting of the isolates, particularly cluster isolates, was not performed by MIRU-VNTR typing or by more discriminatory WGS or cgMLST.

## Conclusion

In conclusion, our data show that rapid molecular diagnostic tests that interrogate hot spot region of *rpoB* and *katG315* + *inhA*-RR will have a sensitivity of 98% for rifampicin resistance detection, 98% for isoniazid resistance detection and 96% for MDR-TB in Kuwait. Our data also showed that 53 of 93 (57%) MDR-TB strains contain *pncA* mutations and are thus additionally resistant to pyrazinamide. Our study provides first insight into molecular epidemiology of MDR-TB in Kuwait and identified at least five clusters of local transmission of MDR-TB involving 2–6 subjects which had escaped detection by routine surveillance studies. Prospective detection of resistance-conferring mutations in major gene targets and molecular fingerprinting studies can identify possible cases of local transmission of MDR-TB in low MDR-TB settings which can then be investigated for epidemiological linkage and contact tracing to arrest further transmission of infection.

## Supplementary information

**Additional file 1: Table S1.** DNA sequences of oligonucleotide primers used for the amplification of various gene targets and the size of amplicon obtained for each primer pair.

**Additional file 2: Table S2.** DNA sequences of oligonucleotide primers used for the sequencing of various gene targets.

## Data Availability

The datasets used and/or analyzed during the current study are available from the corresponding author on reasonable request.

## Abbreviations

DST: drug susceptibility testing
DR-TB: drug-resistant tuberculosis
*inhA*-RR: *inhA*-regulatory region
*katG315*: *katG* codon 315
MDR-TB: multidrug-resistant tuberculosis
MGIT: mycobacteria growth indicator tube
MLSA: multi-locus sequence analysis
cgMLST: core genome multi-locus sequence typing
MIRU-VNTR: mycobacterial interspersed repetitive unit-variable-number tandem repeat
PZA: pyrazinamide
SIT: shared international type
UPGMA: unweighted pair group method with arithmetic averages
XDR-TB: extensively drug-resistant tuberculosis
WGS: whole genome sequencing
WHO: World Health Organization

## References

[1] World Health Organization (2018). Global tuberculosis report 2018. WHO/CDS/TB/2018.20.

[2] Ahmad S (2010). New approaches in the diagnosis and treatment of latent tuberculosis infection. Respir Res.

[3] Pareek M, Greenaway C, Noori T, Munoz J, Zenner D (2016). The impact of migration on tuberculosis epidemiology and control in high-income countries: a review. BMC Med.

[4] Yates TA, Khan PY, Knight GM, Taylor JG, McHugh TD, Lipman M (2016). The transmission of *Mycobacterium tuberculosis* in high burden settings. Lancet Infect Dis.

[5] Ahmad S, Mokaddas E (2014). Current status and future trends in the diagnosis and treatment of drug-susceptible and multidrug-resistant tuberculosis. J Infect Pub Health.

[6] Dheda K, Chang KC, Guglielmetti L, Furin J, Schaaf HS, Chesov D (2017). Clinical management of adults and children with multidrug-resistant and extensively drug-resistant tuberculosis. Clin Microbiol Infect.

[7] Miotto P, Zhang Y, Cirillo DM, Yam WC (2018). Drug resistance mechanisms and drug susceptibility testing for tuberculosis. Respirology.

[8] Schön T, Miotto P, Köser CU, Viveiros M, Böttger E, Cambau E (2017). *Mycobacterium tuberculosis* drug-resistance testing: challenges, recent developments and perspectives. Clin Microbiol Infect.

[9] Allix-Béguec C, Arandjelovic I, Bi L, Beckert P, Bonnet M, CRyPTIC Consortium and the 100,000 Genomes Project (2018). Prediction of susceptibility to first-line tuberculosis drugs by DNA sequencing. N Engl J Med.

[10] Zignol M, Cabibbe AM, Dean AS, Glaziou P, Alikhanova N, Ama C (2018). Genetic sequencing for surveillance of drug resistance in tuberculosis in highly endemic countries: a multi-country population-based surveillance study. Lancet Infect Dis.

[11] Miotto P, Cabibbe AM, Borroni E, Degano M, Cirillo DM (2018). Role of disputed mutations in the *rpoB* gene in interpretation of automated liquid MGIT culture results for rifampin susceptibility testing of *Mycobacterium tuberculosis*. J Clin Microbiol.

[12] Al-Mutairi NM, Ahmad S, Mokaddas E, Eldeen HS, Joseph S (2019). Occurrence of disputed *rpoB* mutations among *Mycobacterium tuberculosis* isolates phenotypically susceptible to rifampicin in a country with a low incidence of multidrug-resistant tuberculosis. BMC Infect Dis.

[13] Jajou R, van der Laan T, de Zwaan R, Kamst M, Mulder A, de Neeling A (2019). WGS more accurately predicts susceptibility of Mycobacterium tuberculosis to first-line drugs than phenotypic testing. J Antimicrob Chemother.

[14] Mokaddas E, Ahmad S, Samir I (2008). Secular trends in susceptibility patterns of *Mycobacterium tuberculosis* isolates in Kuwait, 1996–2005. Int J Tuberc Lung Dis.

[15] Ahmad S, Mokaddas E, Al-Mutairi NM (2018). Epidemiology of tuberculosis and multidrug-resistant tuberculosis in the Middle East Region. Expert Rev Anti Infective Ther.

[16] Abal AT, Ahmad S, Mokaddas E (2002). Variations in the occurrence of the S315T mutation within the *katG* gene in isoniazid-resistant clinical *Mycobacterium tuberculosis* isolates from Kuwait. Microb Drug Resist.

[17] Ahmad S, Mokaddas E, Fares E (2002). Characterization of *rpoB* mutations in rifampin-resistant *Mycobacterium tuberculosis* isolates from Kuwait and Dubai. Diagn Microbiol Infect Dis.

[18] Ahmad S, Mokaddas E (2005). The occurrence of rare *rpoB* mutations in rifampicin-resistant *Mycobacterium tuberculosis* isolates from Kuwait. Int J Antimicrob Agents.

[19] Ahmad S, Al-Mutairi NM, Mokaddas E (2012). Variations in the occurrence of specific *rpoB* mutations in rifampicin-resistant *Mycobacterium tuberculosis* strains isolated from patients of different ethnic groups in Kuwait. Indian J Med Res.

[20] Mokaddas E, Ahmad S, Abal AT, Al-Shami AS (2005). Molecular fingerprinting reveals familial transmission of rifampin-resistant tuberculosis in Kuwait. Ann Saudi Med.

[21] Spies FS, Ribeiro AW, Ramos DF, Ribeiro MO, Martin A, Palomino JC (2011). Streptomycin resistance and lineage-specific polymorphisms in *Mycobacterium tuberculosis gidB* gene. J Clin Microbiol.

[22] Ramirez-Busby SM, Rodwell TC, Fink L, Catanzaro D, Jackson RL, Pettigrove M (2017). Multinational analysis of mutations and heterogeneity in PZase, RpsA, and PanD associated with pyrazinamide resistance in M/XDR *Mycobacterium tuberculosis*. Sci Rep.

[23] Ahmad S, Mokaddas E, Al-Mutairi N, Eldeen HS, Mohammadi S (2016). Discordance across phenotypic and molecular methods for drug susceptibility testing of drug-resistant *Mycobacterium tuberculosis* isolates in a low TB incidence country. PLoS ONE.

[24] Mokaddas E, Ahmad S (2007). Development and evaluation of a multiplex PCR for rapid detection and differentiation of *Mycobacterium tuberculosis* complex members from non-tuberculous mycobacteria. Jap J Infect Dis..

[25] Ahmad S, Fares E, Araj GF, Chugh TD, Mustafa AS (2002). Prevalence of S315T mutation within the *katG* gene in isoniazid-resistant clinical *Mycobacterium tuberculosis* isolates from Dubai and Beirut. Int J Tuberc Lung Dis.

[26] Al-Mutairi N, Ahmad S, Mokaddas E (2011). Performance comparison of four methods for rapid detection of multidrug-resistant *Mycobacterium tuberculosis* strains. Int J Tuberc Lung Dis.

[27] Ahmad S, Jaber AA, Mokaddas E (2007). Frequency of *embB* codon 306 mutations in ethambutol-susceptible and -resistant clinical *Mycobacterium tuberculosis* isolates in Kuwait. Tuberculosis (Edinburgh).

[28] Al-Mutairi N, Ahmad S, Mokaddas E (2018). Molecular screening versus phenotypic susceptibility testing of multidrug-resistant *Mycobacterium tuberculosis* isolates for streptomycin and ethambutol. Microb Drug Resist..

[29] Miotto P, Cabibbe AM, Feuerriegel S, Casali N, Drobniewski F, Rodionova Y (2014). *Mycobacterium tuberculosis* pyrazinamide resistance determinants: a multicenter study. MBio.

[30] Kamerbeek J, Schouls L, Kolk A, van Agterveld M, van Soolingen D, Kuijper S (1997). Simultaneous detection and strain differentiation of *Mycobacterium tuberculosis* for diagnosis and epidemiology. J Clin Microbiol.

[31] Couvin D, David A, Zozio T, Rastogi N (2018). Macro-geographical specificities of the prevailing tuberculosis epidemic as seen through SITVIT2, an updated version of the *Mycobacterium tuberculosis* genotyping database. Infect Genet Evol.

[32] Feuerriegel S, Köser CU, Niemann S (2014). Phylogenetic polymorphisms in antibiotic resistance genes of the *Mycobacterium tuberculosis* complex. J Antimicrob Chemother.

[33] World Health Organization (2012). Recommendations for investigating contacts of persons with infectious tuberculosis in low- and middle-income countries.

[34] Van Soolingen D (2001). Molecular epidemiology of tuberculosis and other mycobacterial infections: main methodologies and achievements. J Int Med.

[35] Gauthier M, Bidault F, Mosnier A, Bablishvili N, Tukvadze N, Somphavong S (2015). High-throughput mycobacterial interspersed repetitive-unit-variable-number tandem-repeat genotyping for *Mycobacterium tuberculosis* epidemiological studies. J Clin Microbiol.

[36] Merker M, Kohl TA, Niemann S, Supply P (2017). The evolution of strain typing in the *Mycobacterium tuberculosis* complex. Adv Exp Med Biol.

[37] de Viedma D, Pérez-Lago L (2018). The evolution of genotyping strategies to detect, analyze, and control transmission of tuberculosis. Microbiol Spectr.

[38] Meehan CJ, Moris P, Kohl TA, Pečerska J, Akter S, Merker M (2018). The relationship between transmission time and clustering methods in *Mycobacterium tuberculosis* epidemiology. EBioMedicine.

[39] Kohl TA, Harmsen D, Rothgänger J, Walker T, Diel R, Niemann S (2018). Harmonized genome wide typing of tubercle bacilli using a web-based gene-by-gene nomenclature system. EBioMedicine.

[40] Liu CH, Li HM, Lu N, Wang Q, Hu YL, Yang X (2012). Genomic sequence based scanning for drug resistance-associated mutations and evolutionary analysis of multidrug-resistant and extensively drug-resistant *Mycobacterium tuberculosis*. J Infect.

[41] Ahmad S, Mokaddas E (2009). Recent advances in the diagnosis and treatment of multidrug-resistant tuberculosis. Respir Med.

[42] Campbell PJ, Morlock GP, Sikes RD, Dalton TL, Metchock B, Starks AM (2011). Molecular detection of mutations associated with first- and second-line drug resistance compared with conventional drug susceptibility testing of *Mycobacterium tuberculosis*. Antimicrob Agents Chemother.

[43] Rodwell TC, Valafar F, Douglas J, Qian L, Garfein RS, Chawla A (2014). Predicting extensively drug-resistant *Mycobacterium tuberculosis* phenotypes with genetic mutations. J Clin Microbiol.

[44] Vilchèze C, Jacobs WR. Resistance to isoniazid and ethionamide in *Mycobacterium tuberculosis*: genes, mutations, and causalities. Microbiol Spectrum. 2014. p. 2. 10.1128/microbiolspec.MGM2-0014-2013 PMC6636829 26104204

[45] Gagneux S, Long CD, Small PM, Van T, Schoolnik GK, Bohannan BJ (2006). The competitive cost of antibiotic resistance in *Mycobacterium tuberculosis*. Science.

[46] Ahmad S, Mokaddas E (2018). Recent advances in proper management of multidrug-resistant tuberculosis. Kuw Med J.

[47] Chedore P, Bertucci L, Wolfe J, Sharma M, Jamieson F (2010). Potential for erroneous results indicating resistance when using the Bactec MGIT 960 system for testing susceptibility of *Mycobacterium tuberculosis* to pyrazinamide. J Clin Microbiol.

[48] Hoffner S, Angeby K, Sturegård E, Jönsson B, Johansson A, Sellin M (2013). Proficiency of drug susceptibility testing of *Mycobacterium tuberculosis* against pyrazinamide: the Swedish experience. Int J Tuberc Lung Dis..

[49] Piersimoni C, Mustazzolu A, Giannoni F, Bornigia S, Gherardi G, Fattorini L (2013). Prevention of false resistance results obtained in testing the susceptibility of *Mycobacterium tuberculosis* to pyrazinamide with the Bactec MGIT 960 system using a reduced inoculum. J Clin Microbiol.

[50] Pang Y, Zhu D, Zheng H, Shen J, Hu Y, Liu J (2017). Prevalence and molecular characterization of pyrazinamide resistance among multidrug-resistant *Mycobacterium tuberculosis* isolates from Southern China. BMC Infect Dis.

[51] Zhang Y, Chiu Chang K, Leung CC, Wai Yew W, Gicquel B, Fallows D (2012). ‘Z^s^-MDR-TB’ versus ‘Z^r^-MDR-TB’: improving treatment of MDR-TB by identifying pyrazinamide susceptibility. Emerg Microbes Infect.

[52] Hanekom M, van Pittius NC, McEvoy C, Victor TC, Van Helden PD, Warren RM (2011). *Mycobacterium tuberculosis* Beijing genotype: a template for success. Tuberculosis (Edinb).

[53] Hoffner S, Sahebi L, Ansarin K, Sabour S, Mohajeri P (2018). *Mycobacterium tuberculosis* of the Beijing genotype in Iran and the World Health Organization Eastern Mediterranean Region: a meta-analysis. Microb Drug Resist.

[54] Varghese B, Supply P, Allix-Béguec C, Shoukri M, Al-Omari R, Herbawi M (2013). Admixed phylogenetic distribution of drug resistant *Mycobacterium tuberculosis* in Saudi Arabia. PLoS ONE.

[55] Poudel A, Maharjan B, Nakajima C, Fukushima Y, Pandey BD, Beneke A (2013). Characterization of extensively drug-resistant *Mycobacterium tuberculosis* in Nepal. Tuberculosis (Edinburgh).

[56] Chen H, He L, Huang H, Shi C, Ni X, Dai G (2017). *Mycobacterium tuberculosis* lineage distribution in Xinjiang and Gansu Provinces. China. Sci Rep.

[57] Nikolayevskyy V, Niemann S, Anthony R, van Soolingen D, Tagliani E, Ködmön C (2019). Role and value of whole genome sequencing in studying tuberculosis transmission. Clin Microbiol Infect.

[58] Appelgren A, Morquin D, Dufour S, Le Moing V, Reynes J, Lotthé A (2017). Investigation of pre-XDR Beijing *Mycobacterium tuberculosis* transmission to a healthcare worker in France, 2016. J Hosp Infect.

[59] Walker TM, Lalor MK, Broda A, Ortega LS, Morgan M, Parker L (2014). Assessment of *Mycobacterium tuberculosis* transmission in Oxfordshire, UK, 2007–12, with whole pathogen genome sequences: an observational study. Lancet Respir Med.

[60] Mathema B, Andrews JR, Cohen T, Borgdorff MW, Behr M, Glynn JR (2017). Drivers of tuberculosis transmission. J Infect Dis.

[61] Al-Ghafli H, Kohl TA, Merker M, Varghese B, Halees A, Niemann S (2018). Drug-resistance profiling and transmission dynamics of multidrug-resistant *Mycobacterium tuberculosis* in Saudi Arabia revealed by whole genome sequencing. Infect Drug Resist.

[62] Lalor MK, Casali N, Walker TM, Anderson LF, Davidson JA, Ratna N (2018). The use of whole-genome sequencing in cluster investigation of a multidrug-resistant tuberculosis outbreak. Eur Respir J.

