# Correction to: Molecular characterization of multidrug-resistant *Mycobacterium tuberculosis* (MDR-TB) isolates identifies local transmission of infection in Kuwait, a country with a low incidence of TB and MDR-TB

**DOI:** 10.1186/s40001-020-00412-7

**Published:** 2020-04-21

**Authors:** Noura M. Al-Mutairi, Suhail Ahmad, Eiman M. Mokaddas

**Affiliations:** Department of Microbiology, Faculty of Medicine, Kuwait University, P. O. Box 24923, 13110 Safat, Kuwait

## Correction to: Eur J Med Res (2019) 24:38 10.1186/s40001-019-0397-2

The original publication of this article [1] contained few erroneous paragraphs and errors in Table 1 and Table 2. The first four paragraphs are in the ‘Results’ section while the last four paragraphs are in the ‘Discussion’ section. The errors in Table 1 involve the number of isolates tested for pyrazinamide and pyrazinamide susceptible isolates, ethambutol-susceptible isolates with a mutation and number of resistant isolates with a mutation for streptomycin. The error in Table 2 involves wrong codon number for a mutation in isolate KM17-01 in Cluster XII for *gidB* gene. The updated informations have been indicated in **bold** and also refer corrected Tables 1 and 2.

**Table 1:** Phenotypic resistance by MGIT 960 system to anti-TB drugs among 93 multidrug-resistant *M. tuberculosis* isolates and number of susceptible and resistant isolates with mutations in target genes for each drug

Anti-tuberculosis drug	No. of isolates tested	No. of susceptible isolates	No. of susceptible isolates with mutation^a^	No. of resistant isolates	No. (%) of resistant isolates with mutation^a^
Rifampicin	93	0	0	93	93 (100)
Isoniazid	93	0	0	93	92 (98.9)
Pyrazinamide	**46**	**10**	0	36	30 (83.3)
Ethambutol	93	52	**39**^b^	41	38 (92.7)
Streptomycin	93	34	0	59	**51 (86.4)**

^a^Resistance conferring mutations were detected in *rpoB* for rifampicin, *katG* + *inhA* for isoniazid, *pncA* for pyrazinamide, *embB* for ethambutol, and *rpsL* + rrs for streptomycin

^b^*M. tuberculosis* isolates with *embB* mutations usually confer low level of resistance to ethambutol which are often missed by the MGIT 960 system [23, 28]

**Table 2:** Detailed clinical, demographic and molecular characteristics of 42 *M. tuberculosis* isolates in 16 (Cluster I to Cluster XVI) clusters

Cluster no.	Clinica specimen	Isolate no.	Year of isolation	Patient’s nationality	Spoligotyping data		Genetic alteration detected in								
					SIT	Mtb family	*rpoB*	*katG*	*inhA*	*pncA*	*embB*	*rpsL*	*rrs*	*gidB*	*rpsA*
I	Sputum	KM06-153	2006	Indian	255	Beijing	TCG456TTG	ACG315ACC	WT	WT	ATG306GTG	AAG43AGG	WT	N. D.	N. D.
	CSF	KM09-22	2009	Indian	255	Beijing	TCG456TTG	ACG315ACC	WT	WT	ATG306GTG	AAG43AGG	WT	N. D.	N. D.
	Sputum	KM13-37	2013	Indian	1	Beijing	TCG456TTG	ACG315ACC	WT	WT	ATG306GTG	AAG43AGG	WT	N. D.	N. D.
	FNA	KM16-06	2016	Nepalese	1	Beijing	TCG456TTG	ACG315ACC	WT	WT	ATG306GTG	AAG43AGG	WT	N. D.	N. D.
	FNA	KM17-03	2017	Indian	1	Beijing	TCG456TTG	ACG315ACC	WT	WT	ATG306GTG	AAG43AGG	WT	N. D.	N. D.
II	Sputum	KM14-58	2014	Nepalese	1	Beijing	TCG456TTG	ACG315ACC	WT	GTG139GCG	ATG306GTG	AAG43AGG	WT	GAA92GAC + *GCA205GCG*	*CGA212CGC*
	Sputum	KM14-69	2014	Indian	1	Beijing	TCG456TTG	ACG315ACC	WT	GTG139GCG	ATG306GTG	AAG43AGG	WT	*GCA205GCG*	WT
III	Sputum	KM08-501	2008	Kuwaiti	1	Beijing	TCG456TTG	ACG315ACC	WT	GGT139GTT	ATG306GTG	AAG43AGG	WT	GAA92GAC + *GCA205GCG*	*CGA212CGC*
	Sputum	KM08-502	2008	Kuwaiti	1	Beijing	TCG456TTG	ACG315ACC	WT	GGT139GTT	ATG306GTG	AAG43AGG	WT	GAA92GAC + *GCA205GCG*	*CGA212CGC*
	Sputum	KM09-207	2009	Indian	1	Beijing	TCG456TTG	ACG315ACC	WT	GGT139GTT	ATG306GTG	AAG43AGG	WT	GAA92GAC + *GCA205GCG*	*CGA212CGC*
IV	Sputum	KM12-05	2012	Ethiopian	21	CAS1-Kili	TCG456TTG	ACG315ACC	WT	Ins193A (FS) + *TCC65TCT*	ATG306GTG	AAG88AGG	WT	N. D.	N. D.
	Sputum	KM12-17	2012	Ethiopian	1144	T1	TCG456TTG	ACG315ACC	WT	Ins193A (FS) + *TCC65TCT*	ATG306GTG	AAG88AGG	WT	N. D.	N. D.
	Sputum	KM15-08	2015	Ethiopian	21	CAS1-Kili	TCG456TTG	ACG315ACC	WT	Ins193A (FS) + *TCC65TCT*	ATG306GTG	AAG88AGG	WT	N. D.	N. D.
V	Sputum	KM07-333	2007	Indonesian	Orphan	N. A.	TCG456TTG	ACG315ACC	WT	WT	WT	WT	WT	N. D.	N. D.
	Sputum	KM10-23	2010	Indian	355	EAI3-IND	TCG456TTG	ACG315ACC	WT	WT	WT	WT	WT	N. D.	N. D.
VI	Sputum	KM07-293	2007	Filipino	194	LAM2	TCG456TTG	ACG315ACC	WT	WT	CAG497CGG	WT	WT	N. D.	N. D.
	Sputum	KM12-01	2012	Filipino	25	CAS1-Delhi	TCG456TTG	ACG315ACC	WT	WT	CAG497CGG	WT	WT	N. D.	N. D.
VII	Sputum	KM09-202	2009	Ethiopian	47	H1	GTC176TTC	ACG315ACC	WT	WT	WT	WT	WT	N. D.	N. D.
	Sputum	KM15-17	2015	Indian	47	H1	GTC176TTC	ACG315ACC	WT	WT	WT	WT	WT	N. D.	N. D.
VIII	Sputum	KM14-67	2014	Ethiopian	149	T3-ETH	TCG456TTG	ACG315ACC	WT	− 11 A/G	ATG306ATC	WT	WT	GGT69GAT	WT
	Sputum	KM15-21	2015	Ethiopian	149	T3-ETH	TCG456TTG	ACG315ACC	WT	− 11 A/G	ATG306ATC	WT	WT	GGT69GAT	WT
IX	Sputum	KM07-283	2007	Indian	26	CAS1-Delhi	TCG456TTG	ACG315ACC	WT	*TCC65TCT*	ATG306ATA	WT	WT	N. D.	N. D.
	Sputum	KM14-68	2014	Indian	Orphan	N. A.	TCG456TTG	ACG315ACC	WT	*TCC65TCT*	ATG306ATA	WT	WT	N. D.	N. D.
	Sputum	KM17-20	2017	Kuwaiti	1	Beijing	TCG456TTG	ACG315ACC	WT	− 11 A/G	CAG497CGG	AAG43AGG	WT	GAA92GAC + *GCA205GCG*	WT
X	Sputum	KM17-22	2017	Kuwaiti	1	Beijing	TCG456TTG	ACG315ACC	WT	− 11 A/G	CAG497CGG	AAG43AGG	WT	GAA92GAC + *GCA205GCG*	*CGA212CGC*
	Sputum	KM17-73	2017	Indian	1	Beijing	TCG456TTG	ACG315ACC	WT	− 11 A/G	CAG497CGG	AAG43AGG	WT	GAA92GAC + *GCA205GCG*	*CGA212CGC*
	Pus	KM11-503	2011	Kuwaiti	1	Beijing	TCG456TTG	ACG315ACC	WT	− 11 A/G	GGC406GAC	AAG43AGG	WT	GAA92GAC + GCA205GCG	*CGA212CGC*
	Sputum	KM14-56	2014	Kuwaiti	1	Beijing	TCG456TTG	ACG315ACC	WT	− 11 A/G	GGC406GAC	AAG43AGG	WT	GAA92GAC + GCA205GCG	*CGA212CGC*
XI	Sputum	KM15-13	2015	Kuwaiti	1	Beijing	TCG456TTG	ACG315ACC	WT	− 11 A/G	GGC406GAC	AAG43AGG	WT	GAA92GAC + GCA205GCG	*CGA212CGC*
	Sputum	KM15-26	2015	Kuwaiti	1	Beijing	TCG456TTG	ACG315ACC	WT	− 11 A/G	GGC406GAC	AAG43AGG	WT	GAA92GAC + *GCA205GCG*	*CGA212CGC*
	Sputum	KM17-02	2015	Kuwaiti	1	Beijing	TCG456TTG	ACG315ACC	WT	− 11 A/G	GGC406GAC	AAG43AGG	WT	GAA92GAC + *GCA205GCG*	*CGA212CGC*
	Sputum	KM17-69	2017	Kuwaiti	1	Beijing	TCG456TTG	ACG315ACC	WT	− 11 A/G	GGC406GAC	AAG43AGG	WT	GAA92GAC + *GCA205GCG*	*CGA212CGC*
XII	Sputum	KM16-32	2016	Egyptian	19	EAI2-Manila	CAC451TAC	ACG315ACC	− 15 C/T	GAA37AAA	*CTG355CTA* + GAG378GCG	WT	WT	*GTG110GTT* + *GCA205GCG*	WT
	Sputum	KM17-01	2017	Filipino	19	EAI2-Manila	CAC451TAC	ACG315ACC	− 15 C/T	GAA37AAA	*CTG355CTA* + GAG378GCG	WT	WT	**CTC59TTC **+ *GTG110GTT* + *GCA205GCG*	WT
XIII	Pus	KM07-297	2007	Indian	Orphan	N. A.	CAC451GAC	WT	− 15 C/T	*TCC65TCG* + Ins 453T (FS)	ATG306CTG	WT	WT	N. D.	N. D.
	FNA	KM11-502	2015	Indian	3361	T1	CAC451GAC	WT	− 15 C/T	*TCC65TCG* + Ins 453T (FS)	ATG306CTG	WT	WT	N. D.	N. D.
XIV	Sputum	KM06-48	2006	Egyptian	53	T1	TCG456TTG	WT	− 15 C/T	WT	WT	WT	WT	N. D.	N. D.
	Tissue	KM06-277	2006	Filipino	19	EAI2-Manila	TCG456TTG	WT	− 15 C/T	WT	WT	WT	WT	N. D.	N. D.
XV	Sputum	KM16-33	2016	Indian	8	EAI3/EAI5	CAC451TAC	ACG315ACC	WT	CTG35CCG	ATG306GTG + GAG378GCG	AAG43AGG	WT	*GTG110GTT* + *GCA205GCG*	WT
	Sputum	KM17-06	2017	Filipino	8	EAI3/EAI5	CAC451TAC	ACG315ACC	WT	CTG35CCG	ATG306GTG + GAG378GCG	AAG43AGG	WT	*GTG110GTT* + *GCA205GCG*	WT
XVI	Sputum	KM07-231	2007	Indian	Orphan^a^	CAS1-Delhi	ATG440ATA + GAC441TAC	ACG315ACC	WT	*TCC65TCT*	GGC406TGC	WT	WT	*GCA205GCG* + Del 350G (FS)	WT
	Sputum	KM07-252	2007	Syrian	Orphan^a^	CAS1-Delhi	ATG440ATA + GAC441TAC	ACG315ACC	WT	*TCC65TCT*	GGC406TGC	WT	WT	*GCA205GCG* + Del 350G (FS)	WT

Clusters containing MDR-TB strains with identical patterns and isolated within a period of nearly 2 years are shown as underlined. Synonymous mutations are italicized

N. A., not applicable; N. D., not done; CSF, cerebrospinal fluid; FNA, fine needle aspirate; SIT, shared international type; Mtb family, *M. tuberculosis* family; WT, wild-type sequence; Ins, insertion mutation; (FS), frame shift mutation, fine needle aspirate

^a^Both isolates displayed identical spoligotyping pattern

**Incorrect:** Although all 93 MDR-TB isolates were tested for susceptibility to pyrazinamide, only 47 isolates yielded interpretable results; 11 isolates were susceptible and 36 were resistant to this drug including 15 isolates that were resistant to all five drugs. The remaining 46 MDR-TB strains failed to grow at the reduced pH in the absence of the drug.

**Correct:** Although all 93 MDR-TB isolates were tested for susceptibility to pyrazinamide, only **46 isolates** yielded interpretable results; **10 isolates** were susceptible and 36 were resistant to this drug including 15 isolates that were resistant to all five drugs. The remaining **47 MDR-TB strains** failed to grow at the reduced pH in the absence of the drug.

**Incorrect:** The proportion of MDR-TB isolates exhibiting resistance conferring mutations in target genes varied for different anti-TB drugs, being highest for rifampicin and lowest for streptomycin (Table 1).

**Correct:** The proportion of MDR-TB isolates exhibiting resistance conferring mutations in target genes varied for different anti-TB drugs, being highest for rifampicin and lowest for streptomycin **among SIRE drugs** (Table 1).

**Incorrect:** PCR-sequencing of *pncA* identified mutations in 30 of 36 MDR-TB strains phenotypically resistant to pyrazinamide and 23 of 46 isolates for which phenotypic DST data for pyrazinamide was not available while all 11 isolates phenotypically susceptible to pyrazinamide contained wild-type sequence for *pncA*.

**Correct:** PCR-sequencing of *pncA* identified mutations in 30 of 36 MDR-TB strains phenotypically resistant to pyrazinamide and 23 of **47** isolates for which phenotypic DST data for pyrazinamide was not available while all **10** isolates phenotypically susceptible to pyrazinamide contained wild-type sequence for *pncA*.

**Incorrect:** Fifty isolates contained mutations at *embB306* (M306V, n = 28; M306I, n = 19 and M306L, n = 3), 15 isolates contained a mutated *embB406* (G406D, n = 8; G406A, n = 4; G406C, n = 2 and G406S, n = 1), 10 isolates contained a mutated *embB497* (Q497R, n = 6; Q497K, n = 3 and Q497H, n = 1) and one isolate contained a mutation (Y319S) at *embB319*.

**Correct:** Fifty isolates contained mutations at *embB306* (M306V, n = 28; M306I, n = 19 and M306L, n = 3), **16** isolates contained a mutated *embB406* (G406D, n = 8; G406A, **n = 5**; G406C, n = 2 and G406S, n = 1), 10 isolates contained a mutated *embB497* (Q497R, n = 6; Q497K, n = 3 and Q497H, n = 1) and one isolate contained a mutation (Y319S) at *embB319*.

**Incorrect:** Forty-nine of 59 MDR-TB strains additionally resistant to streptomycin contained a mutation in the target genes analysed (Table 1), many of which have been described previously [23, 28]. These included 44 isolates with a mutation in *rpsL* (K43R, n = 33; K43T, n = 1; K88R, n = 5; K88T, n = 4; K88M, n = 1), four isolates with a mutation in *rrs* 500 or 900 region (A514C, n = 1; C517T, n = 1; G878A, n = 1 and A906G, n = 1) and one isolate with *rpsL* K88R + *rrs* C602A double mutation.

**Correct: Fifty-one** of 59 MDR-TB strains additionally resistant to streptomycin contained a mutation in the target genes analysed (Table 1), many of which have been described previously [23, 28]. These included 44 isolates with a mutation in *rpsL* (K43R, n = 33; K43T, n = 1; K88R, n = 5; K88T, n = 4; K88M, n = 1), four isolates with a mutation in *rrs* 500 or 900 region (A514C, n = 1; C517T, n = 1; G878A, n = 1 and A906G, n = 1) and **three isolates with double mutation in*rpsL*and*rrs*genes (*rpsL*K43R + *rrs*C527T, n = 1;*rpsL*K88T + *rrs*C517T, n = 1;*rpsL*K88R + *rrs*C602A, n = 1)**.

**Incorrect:** Resistance conferring mutations in *rpsL* and/or *rrs* gene were detected in majority (49 of 59, 83%) of streptomycin-resistant but not in any streptomycin-susceptible MDR-TB strain while mutations in *embB* gene were detected in both ethambutol-resistant and -susceptible MDR-TB strains, as described in our previous studies [23, 28].

**Correct:** Resistance conferring mutations in *rpsL* and/or *rrs* gene were detected in majority **(51 of 59, 86.4%)** of streptomycin-resistant but not in any streptomycin-susceptible MDR-TB strain while mutations in *embB* gene were detected in both ethambutol-resistant and -susceptible MDR-TB strains, as described in our previous studies [23, 28].

**Incorrect:** Phenotypic DST results for pyrazinamide were available for only 47 of 93 MDR-TB strains while the remaining 46 isolates failed to grow at lower pH. No *pncA* mutation was detected in 50 pansusceptible strains. Analysis of 93 MDR-TB strains showed that 30 of 36 MDR-TB strains phenotypically resistant to pyrazinamide and 23 of 46 isolates for which DST data for pyrazinamide was not available contained a mutation in *pncA* while all 11 MDR-TB strains phenotypically susceptible to pyrazinamide contained wild-type sequence for *pncA*.

**Correct:** Phenotypic DST results for pyrazinamide were available for only **46** of 93 MDR-TB strains while the remaining **47** isolates failed to grow at lower pH. No *pncA* mutation was detected in 50 pansusceptible strains. Analysis of 93 MDR-TB strains showed that 30 of 36 MDR-TB strains phenotypically resistant to pyrazinamide and 23 of **47** isolates for which DST data for pyrazinamide was not available contained a mutation in *pncA* while all **10** MDR-TB strains phenotypically susceptible to pyrazinamide contained wild-type sequence for *pncA*.

**Incorrect:** The two isolates in Cluster XII were also very closely related, with the second isolate (KM17-01) displaying an additional mutation (L95F) in *gidB* which is considered as a hot-spot for mutations in the *M. tuberculosis* genome [21, 57].

**Correct:** The two isolates in Cluster XII were also very closely related, with the second isolate (KM17-01) (Table 2) displaying an additional mutation **(L59F)** in *gidB* which is considered as a hot-spot for mutations in the *M. tuberculosis* genome [21, 57].
