# Supplementary material for: Molecular characterization of multidrug-resistant Mycobacterium tuberculosis (MDR-TB) isolates identifies local transmission of infection in Kuwait, a country with a low incidence of TB and MDR-TB
Source: Eur J Med Res. 2019 Dec 5;24:38. doi: 10.1186/s40001-019-0397-2 (PMC6894303; doi:10.1186/s40001-019-0397-2)
Supplement: Supplementary file 1 — Additional file 1: Table S1. DNA sequences of oligonucleotide primers used for the amplification of various gene targets and the size of amplicon obtained for each primer pair. [file 40001_2019_397_MOESM1_ESM.docx]

**Table S1**. DNA sequences of oligonucleotide primers used for the amplification of various gene targets and the size of amplicon obtained for each primer pair

| Primer | Amplification target | | Direction | Oligonucleotide | Amplicon |
| --- | --- | --- | --- | --- | --- |
| name | gene | region/codon |  | sequence | size (bp) |
| HSRF |  | Hot-spot | Forward | 5’-GACGACATCGACCACTTCGGCAAC-3’ | 426 |
| HSRR |  |  | Reverse | 5’-GAACGGGTTGACCCGCGCGTACA-3’ |  |
| NTF | *rpoB* | N-terminal | Forward | 5'-CGACGAGTGCAAAGACAAGGAC-3' | 310 |
| NTR |  |  | Reverse | 5'-GACGGTGTCGCGCTTGTCGAC-3' |  |
| CL2F |  | Cluster II | Forward | 5'-TCATGGACCAGAACAACCCGCTGT-3' | 679 |
| CL2R |  |  | Reverse | 5'-CGTTGTCGTGCATCACAGTGATGT-3' |  |
| K315F |  | *katG*315AGC | Forward | 5’- CCCATGGCCGCGGCGGTCGACATT-3’ | 355 |
| K315R |  |  | Reverse | 5’- CGCCGTCCTTGGCGGTGTATTGCC-3’ |  |
| KATG1F | *katG* | N-terminal | Forward | 5’-GGTCTATGTCCTGATTGTTCGATA-3’ | 918 |
| KATG1R |  |  | Reverse | 5’-CCATGGGTCTTACCGAAAGTGT-3’ |  |
| KATG2F |  | Middle | Forward | 5’-GAAGTACGGCAAGAAGCTCTCATG-3’ | 787 |
| KATG2R |  |  | Reverse | 5’-CAGCTTGTACCAGGCCTTGGCGAA-3’ |  |
| INHAF |  | Regulatory | Forward | 5’- CGACATACCTGCTGCGCAATTCGT-3’ | 418 |
| INHAR | *inhA* |  | Reverse | 5’- CGTCGCTGTCGGTGACGTCACAT-3’ |  |
| INHAF1 |  | N-terminal | Forward | 5’-TCACACCGACAAACGTCACGAG-3’ | 1287 |
| INHAR1 |  |  | Reverse | 5’-TATGCTTCGATGGCCAAGGCGCTG-3’ |  |
| EMB306A |  | *embB306* & | Forward | 5’-CCGACGCCGTGGTGATATTCGGCT-3’ | 491 |
| EMB306B | *embB* | *embB406* | Reverse | 5’- CAGTGTGAATGCGGCGGTAACGAC-3’ |  |
| EMB497A |  | *embB497* | Forward | 5’-GTCGGCACGTTGCCGTTGGTGT-3’ | 189 |
| EMB497B |  |  | Reverse | 5’-ACCGTCGACGGTGGGCAGGAT-3’ |  |
| PNCF1 |  | N-terminal | Forward | 5`-GCGTCATGGACCCTATATCT-3` | 400 |
| PNCR1 | *pncA* |  | Reverse | 5`-TTCGAAGCCGCTGTACGCTC-3` |  |
| PNCF2 |  | C-terminal | Forward | 5`-TCCATCCCAGTCTGGACACG-3` | 428 |
| PNCR2 |  |  | Reverse | 5`-GCGCGTCACCGGTGAACAAC-3` |  |
| RPSAF1 |  | N-terminal | Forward | 5`-CAATCGGGACCGAGTTTGTCCA-3` | 713 |
| RPSAR1 |  |  | Reverse | 5' -TTATTCAGGAACTCGCTGCGCA-3' |  |
| RPSAF2 | *rpsA* | Middle | Forward | 5’-GGCAAGGAGATCGAGGCCAAGA-3’ | 665 |
| RPSAR2 |  |  | Reverse | 5’-TGCTCGTCGTAACTGTCGGCCA-3’ |  |
| RPSAF3 |  | C-terminal | Forward | 5’-AGGTCATCGACATCGACCTGGA-3’ | 515 |
| RPSAR3 |  |  | Reverse | 5’-ACGATTCCGCCGCATTGCGAGA-3’ |  |
| RPSF | *rpsL* | Entire gene | Forward | 5`-CGCAAGGGTCGTCGGGACAAGA-3` | 422 |
| RPSR |  |  | Reverse | 5`-GGTGACCAACTGCGATCCGTAGA-3` |  |
| 16S1F |  | 500 region | Forward | 5`-GGCCTTCGGGTTGTAAACCTCTT-3` | 276 |
| 16S1R |  |  | Reverse | 5`-GCATTCCACCGCTACACCAGGAA-3` |  |
| 16S2F | *rrs* | 900 region | Forward | 5`-GGATTAGATACCCTGGTAGTCCA-3` | 329 |
| 16S2R |  |  | Reverse | 5`-GGTTGCGCTCGTTGCGGGACTTA-3` |  |
| GIDBF | *gidB* | Entire gene | Forward | 5’-CAGTAAGCGATGCGTGGCCGA-3’ | 914 |
| GIDBR |  |  | Reverse | 5’-CATTCATCGTCGGATTGTGCG-3’ |  |
