# Supplementary material for: Molecular characterization of multidrug-resistant Mycobacterium tuberculosis (MDR-TB) isolates identifies local transmission of infection in Kuwait, a country with a low incidence of TB and MDR-TB
Source: Eur J Med Res. 2019 Dec 5;24:38. doi: 10.1186/s40001-019-0397-2 (PMC6894303; doi:10.1186/s40001-019-0397-2)
Supplement: Supplementary file 2 — Additional file 2: Table S2. DNA sequences of oligonucleotide primers used for the sequencing of various gene targets. [file 40001_2019_397_MOESM2_ESM.docx]

**Table S2**. DNA sequences of oligonucleotide primers used for the sequencing of various gene targets

| Primer | Sequencing target | | Direction | Oligonucleotide sequence |
| --- | --- | --- | --- | --- |
|  | gene | region/codon |  |  |
| HSRFS |  | hot-spot | Forward | 5’- GCATGTCGCGGATGGAGCGGGT- 3’ |
| HSRRS |  |  | Reverse | 5’- GCGTACACCGACAGCGAGCCGA- 3’ |
| NTFS | *rpoB* | N-terminal | Forward | 5'-TTCGTCACCGCCGAGTTCATCAA-3' |
| NTRS |  |  | Reverse | 5'-CTTGACGCTGTGCAGCGTCTTGT-3' |
| CL2FS |  | Cluster II | Forward | 5'-CGCGACGTGCACCCGTCGCACT-3' |
| CL2RS |  |  | Reverse | 5'-GGCACCGCCTGGCGCTGCATGTT-3' |
| K315FS |  | *315AGC* | Forward | 5’-CACACTTTCGGTAAGACCCA -3’ |
| K315RS |  |  | Reverse | 5’-AGCAGGGCTCTTCGTCAGCT-3’ |
| KATG1FS | *katG* | N-terminal | Forward | 5’-GCGATCACATCCGTGATCACA-3’ |
| KATG1RS |  |  | Reverse | 5’-CCATGGGTCTTACCGAAAGTGT-3’ |
| KATG2FS |  | Middle | Forward | 5’-TCATGGGCGGACCTGATTGTTT-3’ |
| KATG2RS |  |  | Reverse | 5’-GTGATCCGCTCATAGATCGGAT-3’ |
| INHAFS |  | Regulatory | Forward | 5’-CAGAAAGGGATCCGTCATGGT-3’ |
| INHARS | *inhA* |  | Reverse | 5’-CAGCCGCTGTGCGATCGCCA-3’ |
| INHAF1 |  | N-terminal | Forward | 5’-TCACACCGACAAACGTCACGAG-3’ |
| INHAFS1 |  |  | Forward | 5’- TGACAGGACTGCTGGACGGCA-3’ |
| INHARS1 |  |  | Reverse | 5’-TACGAATACGCCGAGATGTGGA-3’ |
| EMB306A |  | *embB306* | Forward | 5’-CCGACGCCGTGGTGATATTCGGCT-3’ |
| EMB306B |  |  | Reverse | 5’-CAGTGTGAATGCGGCGGTAACGAC-3’ |
| EMB406A | *embB* | *embB406* | Forward | 5’-TGCTGGCTGCTGCTGTCGCGTGA-3’ |
| EMB406B |  |  | Reverse | 5’-AATGCGGCGGTAACGACGG-3’ |
| EMB497A |  | *embB497* | Forward | 5’-GTCGGCACGTTGCCGTTGGTGT-3’ |
| EMB497B |  |  | Reverse | 5’-ACCGTCGACGGTGGGCAGGAT-3’ |
| PNCFS1 |  | N-terminal | Forward | 5`- GCGTCATGGACCCTATATCT-3` |
| PNCRS1 |  |  | Reverse | 5`-TTCGAAGCCGCTGTACGCTC-3` |
| PNCFS2 | *pncA* | C-terminal | Forward | 5`-TCCATCCCAGTCTGGACACG-3` |
| PNCRS2 |  |  | Reverse | 5`-GCGCGTCACCGGTGAACAAC-3` |
| RPSAFS1 |  | N-terminal | Forward | 5’-GACCGAGTTTGTCCAGCGTGT-3’ |
| RPSARS1 |  |  | Reverse | 5’-TTCAGGAACTCGCTGCGCACCT-3’ |
| RPSAFS2 | *rpsA* | Middle | Forward | 5’-AAGGAGATCGAGGCCAAGATCAT-3’ |
| RPSARS2 |  |  | Reverse | 5’-TCGTAACTGTCGGCCATGCCGTA-3’ |
| RPSAFS3 |  | C-terminal | Forward | 5’-CATCGACATCGACCTGGAGCG-3’ |
| RPSARS3 |  |  | Reverse | 5’-TTCCGCCGCATTGCGAGAACGT-3’ |
| RPSFS | *rpsL* | Entire gene | Forward | 5`-GGACAAGATCAGTAAGGTCAA-3` |
| RPSRS |  |  | Reverse | 5`-CGTTGACCAACGGACGCTTGG-3` |
| 16S1FS |  | 500 | Forward | 5`-ACCTCTTTCACCATCGACGAA-3` |
| 16S1RS |  |  | Reverse | 5`-CAGTACTCTAGTCTGCCCGTAT -3` |
| 16S2FS | *rrs* | 900 | Forward | 5`-CGCCGTAAACGGTGGGTACTA-3` |
| 16S2RS |  |  | Reverse | 5`-GACACGAGCTGACGACAGCCAT-3` |
| GIDBFS | *gidB* | Entire gene | Forward | 5’-CGTCTCGAGAGCGGAGAATGT-3’ |
| GIDBRS |  |  | Reverse | 5’-TGGTGTCATTTCCCGCTGGAA-3’ |
